# Distal Bronchopleural Fistula Closure With Robotic Bronchoscopy

**DOI:** 10.1016/j.atssr.2025.08.004

**Published:** 2025-08-29

**Authors:** Ruth Ackah, Hunter Triplett, Ryan Rimer, Yvonne M. Carter

**Affiliations:** 1 Department of Surgery, The Ohio State University Wexner Medical Center, Columbus, Ohio; 2 Division of Interventional Radiology, University Medical Center of Southern Nevada, Las Vegas, Nevada; 3 Section of Thoracic Surgery, Kirk Kerkorian School of Medicine, University of Nevada, Las Vegas, Nevada

## Abstract

The inability to access distal bronchopleural fistulas with standard bronchoscopes limits the use of endobronchial occlusion techniques. A 43-year-old woman experienced fatigue and cough 1 month after a left lower lobectomy. A chest roentgenogram revealed a new air-fluid level concerning for a bronchopleural fistula. Robotic navigational bronchoscopy identified a small (∼2 mm) opening on the bronchial stump. The fistula was closed by injecting embolic sealant and endovascular coils. Robotic navigational bronchoscopy is an effective tool for localizing distal bronchopleural fistulas, thus allowing for minimally invasive closure techniques.

Predisposing factors such as infection, trauma, radiation, and chemotherapy are associated with bronchopleural fistula (BPF) formation after pulmonary resection. There are multiple options—open window thoracostomy, direct stump closure, and thoracoplasty—in the surgeon’s armamentarium to manage this complication. The decision is invariably dependent on the timing of the occurrence and comorbidities, with conditions such as diabetes mellitus, low body mass index, emphysema, and prolonged intubation associated with higher morbidity and mortality rates.^1^ Catheter-based techniques for BPF closure have been described. Success with this approach is dependent on size and location of the fistula, as well as properties of the devices and sealants.^2^, ^3^, ^4^, ^5^ The inaccessibility of smaller distal fistulas prohibits endobronchial occlusion. Our case describes precise localization of a distal BPF with navigational bronchoscopy for successful catheter-based closure.

A 43-year-old woman with uncontrolled type 1 diabetes mellitus underwent robotic left lower lobectomy for early-stage bronchogenic adenocarcinoma. Her surgical pathologic features were notable for a coccidioidomycosis abscess misdiagnosed as a malignant lesion. She presented 1 month later with a new cough, mild dyspnea, and fatigue. The left pleural cavity was drained with a thoracostomy tube, after a chest roentgenogram revealed a new air-fluid level. Although serous fluid was returned, there was no demonstrable air leak. No obvious fistula was seen on bronchoscopy, but 2 bubbles were noted with saline infusion. A fine-cut (1-mm) chest computed tomographic (CT) scan was obtained. Multiplanar reconstructed images were used to guide the Ion endoluminal robotic bronchoscope (Intuitive Surgical) to an approximately 2-mm opening (Figure 1) along the bronchial staple line. The bronchoscope was replaced with an Apollo Onyx delivery microcatheter (Medtronic). After localization of the fistula was confirmed with the infusion of water-soluble contrast material under fluoroscopy, the catheter was flushed with 0.23 mL dimethyl sulfoxide before injecting 0.46 mL Onyx 34 liquid embolic solution with fluoroscopic guidance. The microcatheter was replaced with a Progreat microcatheter (Terumo; 2.4F; 70^o^) for deployment of Embold endovascular embolization coils (Boston Scientific) and an additional 0.46-mL Onyx solution (Figure 2). No air leak was detected with pressure-controlled ventilation. The upper lobe was fully expanded on follow-up chest roentgenogram (Figure 3). At 8-month follow-up, the patient had no clinical or radiologic evidence of recurrence.

**Figure 1:**
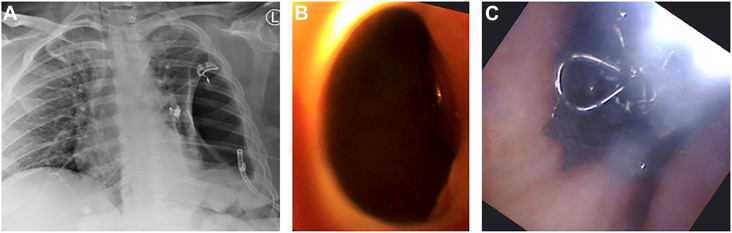
The left upper lobe was partially collapsed on the preoperative chest x-ray (A). The Ion (Intutive) bronchoscope was directed to the opening along the bronchial staple line (B) where a combination of sealant and coils were deployed (C) for closure.

**Figure 2:**
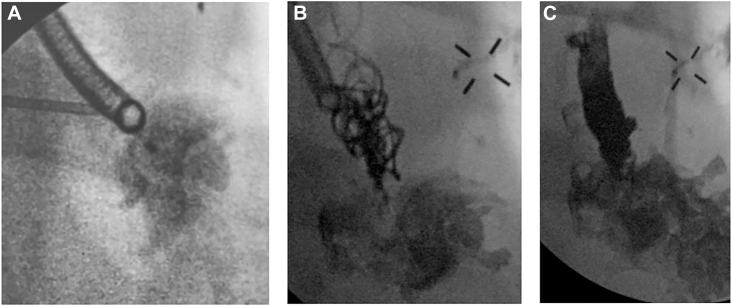
(A) Contrast extravasation into pleural space on fluoroscopy for confirmation, followed by (B) injection of Onyx sealant and (C) endovascular coils.

**Figure 3:**
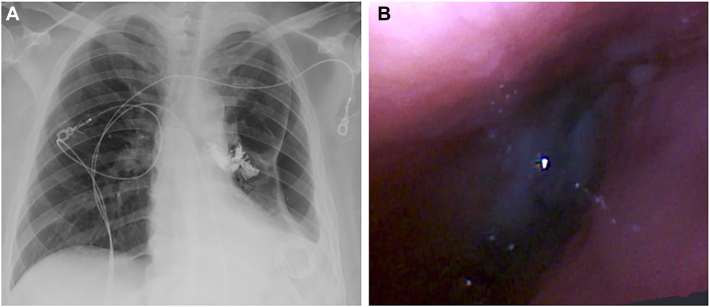
(A) Chest roentgenogram shows a pigtail catheter in a large pleural space with partial upper lobe collapse. (B) Fully expanded upper lobe on a postoperative chest roentgenogram with decreased size residual pleural space.

## Comment

Postresection BPF is a major therapeutic challenge for clinicians. For our patient, successful management was also contingent on sterilization of the associated infection. This complication can be fatal, with mortality rates ranging from 27% to 71%.^1^ The localization of smaller and distal BPFs can be challenging with standard imaging and bronchoscopy. Although helpful, techniques such as saline instillation during bronchoscopy for comprehensive evaluation of the staple line or transthoracic exploration and submersion of the stump under saline are not always conclusive.^6^ In our case, the positive bubble test result without an obvious opening along the bronchial staple line led us to obtain a chest CT scan using the protocol for navigational lung biopsies.

Technical advances in navigational bronchoscopy have made the peripheral tracheobronchial tree accessible. Smaller catheters and flexible instruments have resulted in higher diagnostic yields. The 2 robotic platforms approved for use by the US Food and Drug Administration—the Auris Monarch (Ethicon) and the Ion endoluminal robotic bronchoscopy (Intuitive Surgical)^7^—have primarily been used for endobronchial navigation for lung biopsies. Considering the navigational utility, the indications for this technology are rapidly expanding. This report highlights an early expanded use of the Ion Endoluminal System to identify a distal BPF and catheter-based occlusion.

The principal elements of BPF management include (1) pleural drainage, (2) antimicrobial therapy, (3) optimization of nutrition and management of comorbidities, (4) surgical or endoscopic closure of the fistula, and (5) obliteration of the residual infected space. Larger BPFs are often managed with surgical reexploration, with success rates as high as 90%.^8^ Unfortunately, additional surgery can be a prolonged and morbid experience for some patients. The incidence of secondary complications is higher with protracted treatment, and survival is adversely affected. Time of presentation, dehiscence size, length of the bronchial stump, quality of the remnant stump tissue, presence of residual malignant disease at the stump site, and extent of infectious contamination of the ipsilateral pleural cavity or contralateral lung are all factors to consider in the management of these patients.

Endobronchial therapies have been used early to assist in the spontaneous closure of BPFs after pulmonary resection to avoid additional surgery. Several options have been proposed to occlude smaller (<5-mm) defects, such as fibrin glue and silver nitrate injection.^3^^,^^4^ Larger defects have been managed with polyglycolic acid mesh, endovascular coils, and atrial septal defect occlusive devices.^4^^,^^5^ Similar to the technique described by Watanabe and colleagues,^5^ we combined a sealant with coils, by using the coils as a scaffold to prevent extravasation of the sealant away from the fistula site. The higher viscosity of Onyx made it a more attractive option to control nontarget embolization into the proximal tracheobronchial tree. Our case demonstrates the safe, efficient, and effective use of the robotic endoluminal bronchoscope to identify smaller and distal BPFs. Using catheter-based closure techniques avoids the morbidity and mortality of additional surgery. This report has several limitations, including the anecdotal use of this technique in a single case.

In conclusion, navigational robotic bronchoscopy is an effective tool for rapid, precise localization of small and distal BPFs. Endobronchial identification of these defects allows for minimally invasive occlusion techniques.
